# De novo design of a reversible phosphorylation-dependent switch for membrane targeting

**DOI:** 10.1038/s41467-021-21622-5

**Published:** 2021-03-05

**Authors:** Leon Harrington, Jordan M. Fletcher, Tamara Heermann, Derek N. Woolfson, Petra Schwille

**Affiliations:** 1 Department of Molecular and Cellular Biophysics, Max Planck Institute of Biochemistry, 82152 Martinsried, Germany; 2 School of Chemistry, University of Bristol, Cantock’s Close, Bristol, BS8 1TS UK; 3 School of Biochemistry, University of Bristol, Medical Sciences Building, University Walk, Bristol, BS8 1TD UK; 4 Max Planck-Bristol Centre for Minimal Biology, University of Bristol, Cantock’s Close, Bristol, BS8 1TS UK

**Keywords:** Protein design, Synthetic biology

## Abstract

Modules that switch protein-protein interactions on and off are essential to develop synthetic biology; for example, to construct orthogonal signaling pathways, to control artificial protein structures dynamically, and for protein localization in cells or protocells. In nature, the *E. coli* MinCDE system couples nucleotide-dependent switching of MinD dimerization to membrane targeting to trigger spatiotemporal pattern formation. Here we present a de novo peptide-based molecular switch that toggles reversibly between monomer and dimer in response to phosphorylation and dephosphorylation. In combination with other modules, we construct fusion proteins that couple switching to lipid-membrane targeting by: (i) tethering a ‘cargo’ molecule reversibly to a permanent membrane ‘anchor’; and (ii) creating a ‘membrane-avidity switch’ that mimics the MinD system but operates by reversible phosphorylation. These minimal, de novo molecular switches have potential applications for introducing dynamic processes into designed and engineered proteins to augment functions in living cells and add functionality to protocells.

## Abstract (web summary)

The ability to dynamically control protein-protein interactions and localization of proteins is critical in synthetic biological systems. Here the authors develop a peptide-based molecular switch that regulates dimer formation and lipid membrane targeting via reversible phosphorylation.

## Introduction

The reversible switching of molecular states is ubiquitous in nature, enabling the dynamic regulation of cellular processes essential for life such as cell signaling, transcriptional regulation, and cell division^1^. Such switching may be achieved through conformational change or changes in oligomeric state, which are typically induced by post-translational modification or cofactor binding.

Considerable effort has been made to harness and engineer such natural ‘molecular switches’ in vitro^2–4^. Increasingly, there is a demand to create de novo switches that are orthogonal to existing cellular systems, that enable regulation by different forms of stimuli, and that operate in more-complex environments such as in cells and at membranes. Such artificial molecular switches are important for the development of bioengineering and synthetic biology, as they would allow new regulatory mechanisms and circuits to be incorporated into host cells, or synthetic ‘proto-cells’, endowing their host compartments with new, dynamic functionality^4^.

Examples of engineered molecular switches include those that respond to small-molecule ligands, usually by triggering dimerization of two protein domains^5–7^. Recently, light-operated molecular switches have been designed that allow ‘optogenetic’ control of cellular processes^8^. For example, the LOV2 domain of *Avena sativa* can be harnessed to create photodimerizing domains such as the iLID^9^, TULIP^10^, and LOVTRAP^11^ systems. Exposure to blue light induces a conformational change in the LOV2 domain that is coupled to drive heterodimerization in the case of iLID and TULIP, or dissociation in the case of LOVTRAP. These processes are reversible in the dark.

Another important molecular switch that has been less-extensively pursued is for reversible membrane-targeting, i.e., switching between membrane-bound and solution states. Such switching is common in dynamic, non-linear biological systems as the difference in diffusion coefficients for membrane-bound and cytoplasmic fractions allows a key criterion for reaction-diffusion mechanisms to be met^12^. The ability to tether a molecular cargo to a cellular or artificial membrane reversibly also has important applications in biotechnology and synthetic biology^13,14^.

Natural examples of reversible membrane binding include the *E. coli* MinCDE system^15,16^ and the eukaryotic Rho GTPase Cdc42^17^. In the former, MinD switches repeatedly between a membrane-bound dimer and a cytoplasmic monomer in response to ATP binding and hydrolysis. The membrane affinity of the dimer is enhanced substantially by the avidity effect of bearing two copies of a short, amphipathic membrane-targeting sequence^18,19^. This shuttling between membrane-bound and solution states is an essential aspect of the mechanism that enables the MinCDE system to exhibit spatiotemporal oscillation and pattern formation in vivo and when reconstituted in vitro on artificial membranes^15,16^.

Taking inspiration from natural molecular switches and the *E. coli* Min system, we sought to design a minimal de novo protein–protein interaction (PPI) module that could then be coupled to membrane-targeting peptides to achieve a system that reversibly switches between membrane-bound and solution states in response to a stimulus. We chose protein phosphorylation and dephosphorylation as the stimulus. In nature, reversible protein phosphorylation is a particularly powerful means of dynamically switching PPIs, for example in the regulation of transmembrane receptors^20^. As these modifications are carried out by a large diversity of enzymes (kinases and phosphatases) working in opposition, complex signaling pathways with multiple points of regulation can be formed that respond rapidly to regulatory signals.

Previous design studies have shown that phosphorylation can be harnessed to alter metal-binding properties of small peptides^21,22^, control the assembly of peptide-based hydrogels^23^, and to stabilize peptide oligomers in the phosphorylated state^24,25^, or alternatively destabilize them^26^. The use of small peptide ‘modules’ for the design and engineering of molecular switches is particularly attractive, as many existing systems use larger protein domains the size of which may interfere with the function of fusion domains or cause undesirable trafficking and localization in cellular contexts. However, examples to date have repurposed existing natural motifs, such as Ca^2+^-binding EF hands, bZIP domains, or the Lac repressor tetramerization domain^21,24,25^. To engineer truly orthogonal circuits and enable fully deterministic engineering of PPIs, it is desirable to use synthetic peptide sequences that eliminate any possibility of cross-talk or cryptic function. Indeed, others have described de novo designed peptides and proteins that switch in response to heat^27^, the reduction of disulfide bonds^28^, metal binding^29,30^, and changes in pH^31,32^.

Here, we present a molecular switch based on a de novo designed heterodimeric coiled-coil (CC) assembly^33^, which we engineer to switch dynamically between monomeric and dimeric states by phosphorylation and dephosphorylation. CC peptides are ideal for this purpose, as the rules relating to their folding are relatively well understood, allowing rational design at the amino acid sequence level^34,35^. In addition, stable CC dimers can be achieved with relatively short peptides, enabling the design of minimal, switchable interaction modules. The resulting CC-based interaction modules of our molecular switch can be coupled to membrane-targeting sequences to create a membrane-targeting switch operated by phosphorylation and dephosphorylation. In addition to the important application of dynamically functionalizing membranes, we anticipate that the CC-based molecular switches described here will be highly useful for the engineering of transcriptional regulation and construction of orthogonal, protein-based circuits in both natural and artificial cells^36–40^.

## Results

### Design of CC peptides for molecular switching

**Fig. 1:**
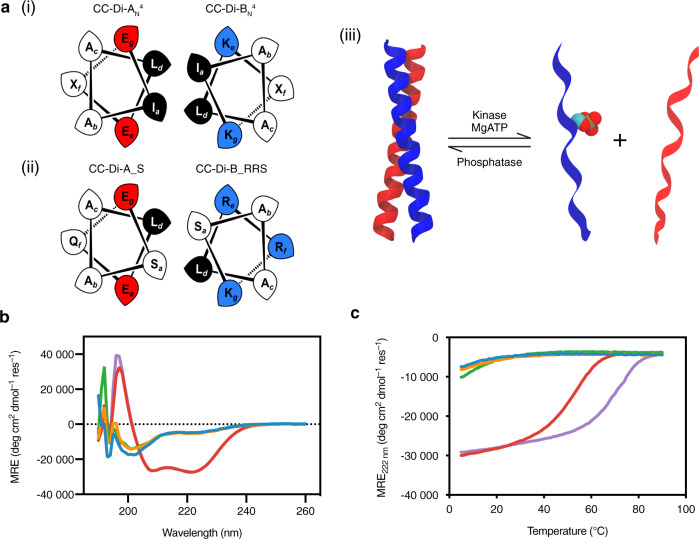
Design and solution-phase characterization of phosphorylation-switchable coiled-coil heterodimers. **a** Helical wheels for the (i) parent and (ii) CC-Di-A_S:CC-Di-B_RRS coiled-coil (CC) designs, and (iii) illustration of the switching concept. In its unphosphorylated state, CC-Di-B_RRS (blue) forms a heterodimeric CC with CC-Di-A_S or CC-Di-A_N_^4^ (red). Phosphorylation of CC-Di-B_RRS by a protein kinase (e.g. PKA) prevents this dimerization due to the bulk and charge of the phosphoryl moiety. Dephosphorylation by a protein phosphatase (e.g. lambda protein phosphatase) re-enables dimerization. **b** CD spectra of individual peptides and their combinations acquired at 25 °C in phosphate-buffered saline (PBS) at pH 7.4: blue, CC-Di-A_S; orange, CC-Di-A_N_^4^; green, CC-Di-B_RRS; red, CC-Di-A_S:CC-Di-B_RRS; and purple, CC-Di-A_N_^4^:CC-Di-B_RRS. **c** Thermal unfolding curves of individual peptides and their combinations in PBS at pH 7.4. The concentration of each peptide was 50 μM in all CD experiments.

For the CC-based design, we began with the well-characterized de novo parallel heterodimeric coiled coil, CC-Di-A_N_^4^B_N_^4^ (Table 1)^33^. CC-Di-A_N_^4^B_N_^4^ exhibits cooperative ‘off-on’ folding and assembly; i.e., the individual peptides remain unfolded and do not form homodimers by themselves (due to interhelical electrostatic repulsion), whereas the two complementary peptides form a characteristically folded coiled-coil heterodimer when mixed. Our aim was to incorporate the recognition motif of the Ser/Thr cAMP-dependent protein kinase (PKA) into just one of the peptides and to complement it with a variant of the other. We hypothesized that phosphorylation of the introduced Ser would disrupt the close packing of the CC interface and prevent dimerization, whereas, with appropriate redesign, the unphosphorylated state should assemble (Fig. 1a).

**Table 1:** Sequences of de novo designed coiled-coil peptides.

The bold letters indicate key residues mutated.

The colour code is to aid visual comparison with the correspondingly coloured elements of Fig. 1.

As PKA is a basophilic kinase, we incorporated the recognition motif (RRXS/T, where X is any amino acid) into the basic peptide (CC-Di-B_N_^4^). The recognition motif was placed such that the phospho-acceptor residue (Ser) was at the ***a*** position of the second heptad repeat (***abcdefg***) of the four-heptad peptide sequence (Fig. 1a and Table 1). We reasoned that: (i) ***a*** sites are in the helix–helix interface and their modification would maximize the impact of phosphorylation to induce CC disassembly; (ii) ***a*** sites of dimeric CCs tolerate substitutions more than the alternative ***d*** sites^41^; and (iii) the polar Arg-Arg residues of the RRXS/T motif would be best accommodated at the ***e*** and ***f*** sites, which are furthest away from the helix-helix interface (Fig. 1a). The resulting peptide is referred to as CC-Di-B_RRS hereafter (Table 1).

Next, we considered appropriate side chains to place at the corresponding ***a*′** position of the acidic partner peptide. In general, it is to be expected that there will be a trade-off between having suffiicient stability for strong ‘off-on’ switching, and sufficient lability to render the switch reversible. The canonical residue at ***a*** in natural coiled coils is Ile^41,42^, and this is used widely in designs including CC-Di-AB^33^. Indeed, the most stabilizing pair at ***a*–*a*′** sites in CC dimers is Ile-Ile^41,42^. Thus, the introduction of the PKA recognition site was already anticipated to destabilize the CC. Energetic analysis confirms that Ser at ***a*** is destabilizing relative to Ile-Ile, and that Ser-Ile is the least destabilizing of the remaining combinations^42^. Considering the next expected ‘least-worst’ combination, Ser when paired with polar residues at ***a′*** prefers the homotypic Ser–Ser pairing^42^. Therefore, to span this range, we prepared two acidic peptides (Table 1): the original parent, CC-Di-A_N_^4^, which has Ile at the ***a*′** site corresponding to the Ser at ***a*** in CC-Di-B_RRS; and CC-Di-A_S, where that Ile is replaced by Ser. These were combined with the basic peptide CC-Di-B_RRS to determine which pairing was optimal for switching.

To allow rapid characterization of our CC modules, the peptides CC-Di-A_N_^4^, CC-Di-A_S, and CC-Di-B_RRS were synthesized by solid-phase peptide synthesis and purified. Circular dichroism (CD) spectroscopy confirmed that the individual peptides were not folded, whereas CD spectra for both CC-Di-A_N_^4^:CC-Di-B_RRS and CC-Di-A_S:CC-Di-B_RRS were characteristic of fully folded α-helical structures, and consistent with heterodimer formation (Fig. 1b). Next, we compared the stabilities of the two hetero-assemblies using thermal unfolding followed by CD spectroscopy (Fig. 1c). As anticipated, the Ser–Ser pairing at ***a–a*′** was less stable than Ile–Ser (*T*_M_ = 54 °C vs 73 °C, respectively), but both combinations were destabilizing relative to the parent heterodimer, CC-Di-A_N_^4^B_N_^4^ (*T*_M_ = 81 °C)^33,42^.

### Phosphorylation-induced switching of the CC modules is stability dependent

**Fig. 2:**
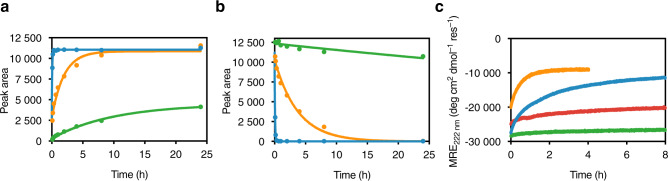
Kinetics of phosphorylation-induced dissociation. Representative phosphorylation reaction time courses for **a** product (CC-Di-B_RRpS) formation, and **b** substrate (CC-Di-B_RRS) consumption, determined by analytical HPLC. Time courses are shown for reactions consisting only of CC-Di-B_RRS (blue); or CC-Di-B_RRS in the presence of CC-Di-A_S (orange) or CC-Di-A_N_^4^ (green). **c** Representative phosphorylation time courses determined by CD spectroscopy for CC-Di-B_RRS in the presence of CC-Di-A_S at 25 °C and 37 °C (blue and orange) and CC-Di-A_N_^4^ at 25 °C and 37 °C (green and red). Each peptide was at 50 µM concentration, and reactions were performed in PBS at pH 7.4 supplemented with 10 mM MgCl_2_ and 1 mM ATP. cAMP-dependent protein kinase (PKA, 8.33 U µL^−1^ of reaction) was added to initiate phosphorylation. Reactions for analytical HPLC were all performed at 25 °C. All reactions were performed in triplicate.

The kinetics of phosphorylation of the CC-Di-B_RRS peptide on its own and when associated with CC-Di-A_S or CC-Di-A_N_^4^ were followed by analytical high-performance liquid chromatography (HPLC), Fig. 2. The phosphorylated form, CC-Di-B_RRpS, eluted before CC-Di-B_RRS due to the presence of the more-polar phosphoserine residue (Supplementary Fig. 1). CC-Di-B_RRS alone was phosphorylated rapidly by PKA, with the reaction reaching completion after 10 min (Fig. 2a, b and Supplementary Fig. 1). The mean rate constant for product formation (*k*_prod_) was 0.20 ± 0.07 min^–1^, and that for substrate consumption (*k*_subs_) was 0.23 ± 0.08 min^–1^ (both *n* = 3; errors reported for mean values are standard deviations throughout unless otherwise indicated). In contrast, phosphorylation of the peptide in the presence of CC-Di-A_S was ≈25–50 times slower: *k*_prod_ = (8.0 ± 0.6) × 10^–3^ min^–1^, and *k*_subs_ = (4.2 ± 0.2) × 10^–3^ min^–1^ (both *n* = 3; *p* < 0.0001). With CC-Di-A_N_^4^ as the partner peptide, phosphorylation was extremely slow (by approximately two to three orders of magnitude compared with the free peptide) and did not reach completion even after 24 h (Fig. 2a, b and Supplementary Fig. 1). As discussed below, these kinetic differences are likely due to differences in temporal accessibility of the phosphorylation site via the unfolded state, and, thus, related to the overall stability of the CC complex.

To complement the HPLC experiments and to characterize the effect of phosphorylation on the assembled CCs, we followed phosphorylation over time by CD spectroscopy monitoring the change in mean residual ellipticity at 222 nm (MRE_222_), which reports directly on helicity. Phosphorylation led to a loss of helicity, suggesting that the CC peptides were indeed switching to disassembled monomeric states. We verified that there was no contribution to the CD signal from the kinase (Supplementary Fig. 2a).

The kinetics of this process were consistent with those derived from the HPLC data, with *k* = (9 ± 2) × 10^–3^ min^–1^ (*n* = 3) for CC-Di-A_S:CC-Di-B_RRS at 25 °C; and *k* = (8 ± 1) × 10^–2^ min^–1^ (*n* = 3) for the same at 37 °C (Fig. 2c and Supplementary Fig. 2b, c). We also performed kinetic CD experiments with triple the amount of PKA. For CC-Di-A_S:CC-Di-B_RRS at 25 °C there was a ≈1.7-fold increase, with *k* = (15 ± 1) × 10^–3^ min^–1^ versus *k* = (9 ± 2) × 10^–3^ min^–1^ (both *n* = 3).

To confirm that the end-point CD spectrum was due to the presence of the phosphorylated product, we prepared CC-Di-B_RRpS by solid-phase synthesis and obtained its CD spectrum with CC-Di-A_S. This was indeed found to be comparable with the end-point spectrum for the phosphorylation reaction of CC-Di-A_S:CC-Di-B_RRS (Supplementary Fig. 3a, b). We also obtained the CD spectrum of CC-Di-B_RRpS in the presence of CC-Di-A_N_^4^, which exhibited some helicity that may be due to formation of a partial CC (Supplementary Fig. 3c).

Finally, we mixed CC-Di-B_RRpS with CC-Di-A_S and measured CD spectra before and after the addition of lambda protein phosphatase (λPP) and MnCl_2_. An increase in helicity was observed after the addition of λPP, consistent with dephosphorylation of CC-Di-B_RRpS and formation of CC-Di-A_S:CC-Di-B_RRS (Supplementary Fig. 4).

In view of the observed differences in phosphorylation kinetics of CC-Di-B_RRS alone and with the different partners, we propose the following reaction scheme.

1 $${\mathrm{A}} - {\mathrm{B}}\mathop { \rightleftharpoons }\limits_{k_{{\mathrm{on}}}}^{k_{{\mathrm{off}}}} A + {\mathrm{B}}\mathop { \to }\limits^{k_{\mathrm{p}}} {\mathrm{A}} + {\mathrm{B}}^\prime$$

Where: A and B are the two CC peptides, and Bʹ is the phosphorylated peptide. This assumes that the kinase recognition site is inaccessible when the peptides are assembled as CCs, and that only the monomeric fraction is modified.

In this scheme, phosphorylation depletes B (CC-Di-B_RRS) shifting the reaction to the right until all B is converted into B′ (CC-Di-B_RRpS). The larger the pool of available B, the faster the initial reaction. This pool is determined by the dissociation constant of A—B. Thus, we expect that the reaction will proceed faster with weaker A–B complexes. This is precisely what we observed: in the presence of CC-Di-A_S the reaction was faster than with CC-Di-A_N_^4^ (Fig. 2). These results suggest that the kinetics of the switching process can be tuned at the amino-acid level by varying the stability of the CC complex, as well as by peptide concentration and temperature. We chose the CC-Di-A_S:CC-Di-B_RRS combination for further use as it exhibited faster kinetics and well-defined monomer/dimer switching compared to CC-Di-A_N_^4^:CC-Di-B_RRS.

### Phosphorylation and dephosphorylation reversibly tethers a cargo molecule to a membrane anchor

**Fig. 3:**
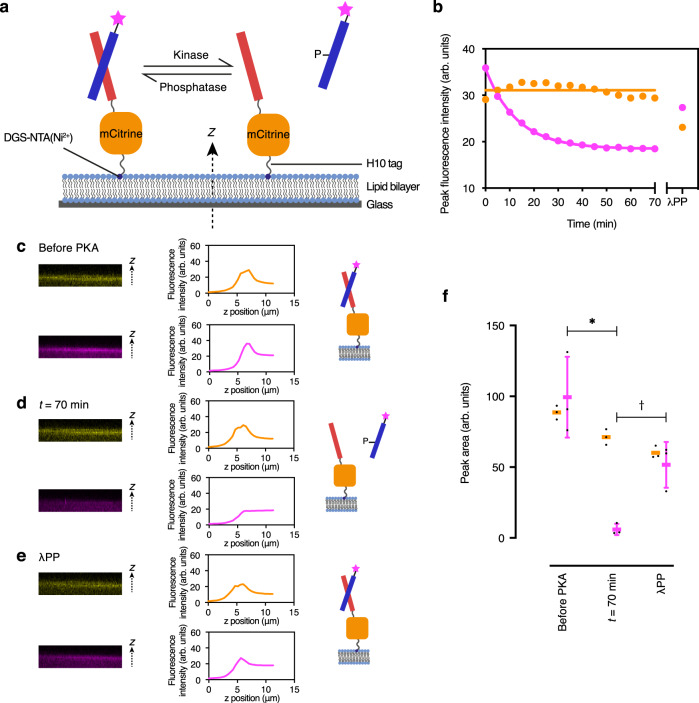
Reversible membrane tethering of a cargo peptide to an anchor protein. **a** Illustration of reversible membrane tethering on Supported Lipid Bilayers (SLBs) with CC-Di-A_S in red, CC-Di-B_RRS in blue, and Alexa Fluor 594 (AF594) in pink. **b** Representative kinetics of phosphorylation-induced release of the cargo peptide (AF594-CC-Di-B_RRS, pink line) from the anchor protein (CC-Di-A_S-mCitrine-H10, orange line). Phosphorylation was initiated with PKA (cAMP-dependent protein kinase) and MgATP and reversed by lambda protein phosphatase (λPP) and MnCl_2_. Experiments were performed in triplicate. Plotted fluorescence intensities (arbitrary units) are peak intensities (i.e., membrane localized), determined from z-axis fluorescence intensity profiles such as those shown in **c**–**e**. Values corresponding to the cargo peptide were fitted with the function $y = y_0 + (y_\infty - y_0)(1 - e^{ - kx})$. Values for the anchor protein were fitted with the function *y* = *k*. Representative xz plane confocal images from z-stacks are shown together with fluorescence z-profiles and illustrative schematics for: **c** CC-Di-A_S-mCitrine-H10 and AF594-CC-Di-B_RRS on an SLB; **d** the system of **c** 70 min after the addition of PKA; **e** the system of **d** after adding λPP. **f** Quantitation of membrane binding of CC-Di-A_S-Citrine-H10 (orange) and AF594-CC-Di-B_RRS (pink) under the conditions of **c**–**e**, calculated from fluorescence z-profiles (*n* = 3 independent experiments; error bars represent mean ± S.D.; *p = 0.005, *t* =  5.6127; ^†^*p* = 0.009, *t* = 4.7414; unpaired two-tailed *t* test). SLBs were DOPC with 2 mol% DGS-NTA(Ni^2+^) and 0.1 mol% ATTO655-DOPE. AF594-CC-Di-B_RRS and CC-Di-A_S-mCitrine-H10 were both at 1 µM. The SLB buffer was 50 mM Tris.HCl, pH 7.5, 300 mM KCl, and 5 mM MgCl_2_.

To use the CC switch for reversible membrane targeting, we began by designing a simple proof-of-concept comprising two components: a permanently attached membrane ‘anchor’, and a ‘cargo’ molecule intended to switch between this anchor and a free-solution state in response to reversible phosphorylation (Fig. 3a). The membrane anchor had three functional modules joined by flexible linkers: an N-terminal CC-Di-A_S as the interaction module, the mCitrine fluorescent protein for visualization, and a C-terminal decahistidine tag for membrane attachment (CC-Di-A_S-mCitrine-H10). The cargo molecule was the synthetic peptide CC-Di-B_RRS N-terminally labeled with a fluorophore (Alexa Fluor 594, giving AF594-CC-Di-B_RRS).

We characterized this system using supported lipid bilayers (SLBs), as these flat, uniform membranes allowed us to quantify membrane localization through fluorescence intensity. The SLBs comprised DOPC doped with Ni-NTA-modified headgroup lipids (DGS-NTA(Ni), for decahistidine attachment) plus ATTO 655-labeled DOPE (for imaging the lipid bilayer). After passivating the SLBs with BSA, they were incubated with the anchor fusion protein for 30 min^43^. Membrane localization was confirmed by z-stacked confocal microscope images through the lipid bilayer (Fig. 3a), which showed coincident peaks of fluorescence intensity from the anchor protein and the labeled lipids (Supplementary Fig. 5).

Next, the cargo peptide was added to the solution in the unphosphorylated state. A peak of fluorescence intensity from the cargo colocalized with those from the anchor protein and the SLB, indicating that the cargo was successfully attached to the membrane (Fig. 3c). To examine detachment of the cargo, and to test that it interacts via CC formation with the anchor, we added PKA and MgATP to the system and monitored the membrane-localized fluorescence intensity of the two components over time. The fluorescence intensity of the cargo at the membrane decreased over time, indicating that the cargo was indeed being released back into solution (Fig. 3b, d), whereas the fluorescence intensity at the membrane from the anchor remained constant over this time.

The change in cargo fluorescence intensity fitted to an exponential decay to give a mean rate constant for membrane detachment of 0.07 ± 0.03 min^–1^ (*n* = 3; Fig. 3b). This is ≈10 times faster than that observed in the HPLC and CD experiments. We posit that this is because of the lower concentrations of anchor and cargo used compared with the peptide concentrations used in the foregoing experiments (1 µM vs 50 µM), which is consistent with a shift in the reaction to the right in our proposed scheme. Of course, other factors such as the presence of fluorophores and other ‘modules’ attached to the CC peptides may also alter the kinetics.

Finally, to confirm reversibility, we added λPP and MnCl_2_ to the SLB system. This caused an increase in fluorescence intensity from the cargo at the membrane, indicating reattachment to the membrane (Fig. 3b, e). The intensity did not completely return to the original value, which we suspected was due to one or more of the following factors: (i) the continued presence of kinase; (ii) dilution by the addition of reagents; (iii) the presence of Mn^2+^ ions; and (iv) degradation/adsorption to surfaces during mixing and liquid handling. However, when the experiments were repeated with the further step of adding a potent PKA inhibitor after dephosphorylation no significant change in membrane binding was observed, indicating that the continued presence of kinase is unlikely to be a contributing factor (Supplementary Fig. 6).

The above membrane-binding switch demonstrates that our CC-based interaction module can be applied to reversibly target molecules to membranes. In its present form, reliance on the non-natural lipid DGS-NTA(Ni) limits application to synthetic cellular mimics; however, it might be possible to implement this mode of membrane targeting in natural cells by using alternative anchoring means, for example, by using phosphoinositide-binding domains or high-affinity membrane-binding peptides.

### Mimicking the MinD switch allows reversible membrane targeting through avidity

We targeted a further membrane-binding switch inspired by the *E. coli* protein MinD. In vivo, MinD forms a cytoplasmic, ADP-bound monomer, and a membrane-associated, ATP-bound dimer. The latter is facilitated by an amphipathic membrane-targeting sequence (MTS) that has a low affinity for lipid membranes. ATP-induced dimerization increases the effective affinity by colocalizing two MTSs, and thus retains MinD at the membrane^18,19^. MinE binds the dimer to enhance ATP hydrolysis, trigger dissociation to monomers, and subsequent membrane detachment. This process cycles to form a reaction-diffusion system that generates striking spatiotemporal oscillations^15^.

**Fig. 4:**
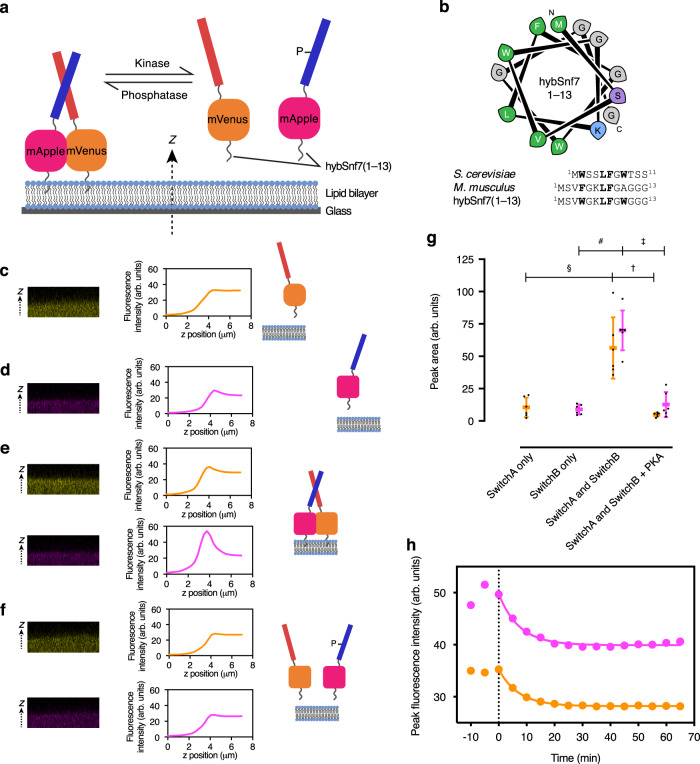
Oligomeric switching of membrane-binding state. **a** Schematic illustrating design and concept of switching with CC-Di-A_S colored red and CC-Di-B_RRS blue. **b** Helical wheel diagram of hybSnf(1–13) and alignment of *S. cerevisiae*, *M. musculus*, and hybrid Snf7 sequences with conserved hydrophobic residues highlighted in bold. **c**–**f** Representative orthogonal views of the xz plane of confocal microscopy image z-stacks with fluorescence intensity z-profiles and illustrative schematics for SLBs with: **c** 1 µM SwitchA only, **d** 1 µM SwitchB only; **e** 1 µM each of SwitchA and SwitchB; and **f** 1 µM each of SwitchA and SwitchB 1 h after MgATP and PKA were added. **g** Quantitation of membrane binding of SwitchA (orange) and SwitchB (pink) under the conditions of panels **c**–**f** calculated from fluorescence z-profiles (*n* = 6 independent experiments; error bars represent mean ± S.D.; ^§^*p* =  0.0011, *t* = 4.5167; ^#^*p* < 0.0001, *t* = 9.4307; ^†^*p* = 0.0003, *t* =  5.3073; ^‡^*p* < 0.0001, *t* = 7.7144; unpaired two-tailed *t* test). **h** Representative kinetics of phosphorylation-induced release of SwitchA and SwitchB from the SLB after the addition of MgATP and PKA at *t* = 0. Plotted fluorescence intensities (arbitrary units) are peak intensities (i.e., membrane localized), determined from z-axis fluorescence intensity profiles such as those shown in **c**–**f**. Values were fitted with the function $y = y_0 + (y_\infty - y_0)(1 - e^{ - kx})$. SLBs were DOPC with 0.1 mol% ATTO655-DOPE. The SLB buffer was 50 mM Tris.HCl, pH 7.5, 300 mM KCl, and 5 mM MgCl_2_. Six experimental replicates were performed.

We reasoned that a de novo designed switch of this type would be powerful and broadly applicable for reversibly targeting proteins to membranes. Further, such a switch would be fully genetically encodable and would therefore be better suited to in vivo applications than the preceding anchor/cargo system described above that relies on non-natural lipids. Therefore, we designed fusion proteins comprising the CC-Di-A_S and CC-Di-B_RRS interaction modules, fluorescent protein labels (mVenus and mApple, respectively), and MTSs (Fig. 4a). Preliminary experiments using the MinD MTS indicated unfavorable electrostatic interactions with the CC modules, which we posit is related to this MTS’s preference for highly negatively charged lipid membrane. Therefore, we sought similar MTSs that rely entirely on amphipathicity rather than electrostatics.

We identified the N-terminal ANCHR domain of the ESCRTIII protein Snf7 as a potential candidate^44^. First, we tested residues 1–13 of Snf7 from *S. cerevisiae*. Unexpectedly, the resulting fusion proteins were truncated during protein expression. A fusion protein with the sequence from *M. musculus* was not cleaved, but did not bind to membranes either, even up to 10 µM concentration. We reasoned that the weakened membrane affinity was due to the absence of tryptophan residues in the MTS motif (Fig. 4b). Therefore, we designed a ‘hybrid’ sequence, hybSnf7(1–13), that combined features of the *S. cerevisiae* and *M. musculus* MTSs (Fig. 4b).

Two fusion proteins were then engineered: hybSnf7(1–13)-mVenus-H6-CC-Di-A_S (hereafter called ‘SwitchA’) and hybSnf7(1–13)-mApple-H6-CC-Di-B_RRS (‘SwitchB’). Full-length proteins were successfully isolated and purified. At 1 µM, both proteins showed negligible membrane localization when added individually to SLBs comprising DOPC doped with 0.1 mol% ATTO655-DOPE (Fig. 4c, d). In contrast, when both proteins were present at 1 µM each, they were strongly localized at the membrane (Fig. 4e), indicative of avidity-enhanced membrane binding by the dimer, as designed. The mVenus fluorescence was diminished in this membrane-bound state, which we attribute to FRET donation to mApple (calculated overlap integral: 4.07 × 10^15^ M^–1^ cm^–1^ nm^4^, *R*_0_ = 60 Å). Indeed, exciting mVenus only gave an increase in fluorescence intensity in the mApple sensitized emission (SE) channel upon heterocomplexation (Supplementary Fig. 7a–c). Acceptor photobleaching of the membrane-bound mApple supported this (Supplementary Fig. 8) to provide further evidence for complexation and a functional switch.

Finally, we tested release of the membrane-bound proteins by switching them to the monomeric states through phosphorylation. Addition of PKA and MgATP led to a time-dependent release of both proteins from the membrane (Fig. 4f, h). Kinetic analysis yielded mean reaction rate constants of 0.10 ± 0.06 min^–1^ for SwitchA and 0.10 ± 0.04 min^–1^ for SwitchB (both *n* = 6). Moreover, the switch could be reversed: addition of λPP and MnCl_2_ increased the membrane localization of both proteins (Supplementary Fig 9), although this was more pronounced for SwitchB than SwitchA. Again, the mApple SE channel diminished upon phosphorylation (and dissociation of the complex), and recovered after addition of λPP and dephosphorylation (Supplementary Fig. 7d–e).

## Discussion

In summary, we have developed a peptide switch based on a de novo coiled-coil heterodimer and operated by phosphorylation and dephosphorylation. We have combined this with natural peptide and protein modules in fusion proteins that function as reversible membrane-targeting switches. Two different modes of membrane targeting are achieved. The first switches a ‘cargo’ fusion protein between solution and membrane-bound states via a permanent membrane-tethered ‘anchor’ protein. The second mimics the membrane switch of the *E. coli* MinD protein^19^, whereby the dimerization of monomers in solution leads to membrane attachment through avidity enhancement of amphipathic membrane-targeting sequences.

To implement mechanisms of biological pattern formation, one next step would be to introduce additional elements to generate feedback loops that control heterodimer association and dissociation. This could recapitulate additional characteristics of the MinCDE system, and thus create systems to generate oscillations or patterns. Also, this type of membrane functionalization has potential applications in biotechnology and synthetic biology. For example, an ‘artificial cytoskeleton’ could be coupled to membranes in cells to serve as scaffolds for biosynthesis^14,45,46^, or used to stabilize and scaffold artificial membranes in protocells^47^.

The de novo nature of our CC-based modules makes them suitable for applications in cells by minimizing the risk of cross-talk or cryptic functions. Equally, their minimal, ground-up construction is well-suited to introducing function in synthetic cellular mimics. The peptide modules are small, comprising 28 residues each, making them relatively unobtrusive as tags in fusion proteins. CCs have proven to be readily designable, as evident from the diversity of different architectures of designed CCs reported in the literature. Therefore, in principle, it should be possible to design similar switches using higher stoichiometries, such as trimers and tetramers^48,49^, which could increase sensitivity and the range of structures into which these switches could be incorporated. Furthermore, because the kinetics of phosphorylation-induced dissociation are governed by the stability of the coiled-coil complex, the rate of dissociation can be tuned, most usefully through mutation or variation of the length of the CC sequences, but also through temperature and peptide concentration.

Aside from the already-discussed use in targeting membranes, we anticipate that our CC-based switching modules will also be useful for the construction of protein-based logic and orthogonal signaling pathways in natural and synthetic cells^40^. This will require the development of additional, orthogonal hetero-oligomeric switch modules incorporating different peptide combinations and kinase/phosphatase recognition sites. Kinase/phosphatase activity might be controlled transcriptionally, by light^50^, or by small-molecules. Protein signaling circuitry and control using protease-based logic gates^39,51^, or degron-coupled protein switches^52,53^ have previously been described. The use of phosphorylation and dephosphorylation as proposed here may offer advantages in that it is a fully reversible and non-destructive post-translational modification, as opposed to proteolytic cleavage or proteasomal degradation. It may also be possible to interface directly with native signaling pathways. Further applications include the controlled assembly or disassembly of other protein complexes to control gene and transcriptional regulation^37,54–56^, and stimulus-responsive disassembly of artificial protein nanostructures^57,58^, for example to release an encapsulated payload. Therefore, the switchable interaction modules described herein have significant potential as components in a variety of bioengineered and artificial systems to introduce dynamics and control.

## Methods

All values are reported as mean ± standard deviation unless otherwise stated. Statistical significance is reported by *p* and *t* values calculated from two-tailed unpaired *t* tests.

All chemicals, reagents, and oligonucleotides were from Sigma Aldrich unless otherwise indicated.

### ATP stock solution

ATP stock solutions were prepared from the ATP disodium salt hydrate and titrated to pH 7.0 with 1 M Tris base. ATP concentration was confirmed spectrophotometrically at 259 nm using an extinction coefficient of 15,400 M^–1^ cm^–1^. Stocks were stored at –80 °C as single-use aliquots.

### Enzymes

cAMP-dependent protein kinase (PKA) and lambda protein phosphatase (λPP) were purchased from New England Biolabs.

### Peptide synthesis and purification

Peptides were synthesized using standard microwave-assisted Fmoc solid-phase protocols on a CEM Liberty Blue automated synthesiser coupled to an inline UV monitor. All peptides were prepared as C-terminal amides at 0.1 mmol scale on Rink Amide ChemMatrix resin. Successive amino acid coupling was achieved using diisopropylcarbodiimide (DIC)/6-chloro-1-hydroxybenzotriazole (Cl-HOBt) coupling protocols, whilst Fmoc-cleavage was performed by treatment with 20% morpholine in dimethyl formamide (DMF). Upon completion, the resin-attached peptide was removed and, where appropriate, the N-terminus acetylated by treatment with 3 eq. acetic anhydride and 4.5 eq *N,N*-diisopropylethylamine (DIPEA) in DMF for 30 min. Owing to its expense, coupling of AlexaFluor 594 carboxylic acid was performed at 5.5 µM scale with 1.1 eq AlexaFluor 594 carboxylic acid, 1 eq. DIC and 1 eq. Cl-HOBt in DMF overnight.

Cleavage from the solid support was performed with a mixture of trifluoroacetic acid (TFA)/triisopropylsilane/water (90/5/5, v/v/v). The cleavage solution was reduced in volume to ∼5 ml by using a flow of nitrogen. Diethyl ether (45 ml) was added to obtain a precipitate that was recovered by refrigerated centrifugation and dissolved in 1:1 acetonitrile:water (10 ml) and lyophilized to yield a white solid. Peptides were purified by reversed-phase HPLC, with a gradient of 20–80% acetonitrile in water (each containing 0.1% TFA) over 45 min on a Vydac TP C18 column (10 µm particle, 22 × 250 mm). Fractions that contained pure peptide were identified by analytical HPLC and matrix-assisted laser desorption/ionization–time of flight (MALDI–TOF) mass spectrometry. Analytical HPLC was performed with a Jasco 2000 series HPLC system with a Phenomenex Kinetex C18 column (5 μm particle, 4.6 × 100 mm), monitored at 220 nm. The gradient was 20–80% acetonitrile/water (each containing 0.1% TFA) over 16 min. MALDI–TOF mass spectra were obtained on a Bruker UltraFlex MALDI–TOF mass spectrometer in positive-ion reflector mode. Peptide solutions were spotted on a ground-steel target plate with dihydroxybenzoic acid as the matrix. Calibration was conducted using the ‘nearest neighbor’ method, with Bruker Peptide Calibration Standard II as the reference masses.

Peptide concentrations were determined in solution by UV absorption spectroscopy using the following extinction coefficients: *ε*_280 nm_ (Trp)  = 5690 M^–1^ cm^–1^, *ε*_280 nm_ (Tyr) = 1280 M^–1^ cm^–1^.

### Plasmids

pT7-CC-Di-A_S-mCitrine-H10 was produced by in vivo homologous recombination^59,60^. The CC-Di-A_S-mCitrine-H10 coding sequence, incorporating flanking 18 bp sequences at 5′ and 3′ ends for homologous recombination into pT7-αHL-D127N between start and stop codons was designed in silico and obtained as synthetic dsDNA from Life Technologies. pT7-αHL-D127N was a kind gift from Prof Hagan Bayley (University of Oxford)^61,62^. pT7-hybSnf7(1–13)-mVenus-H6-CC-Di-A_S and pT7-hybSnf7(1–13)-mApple-H6-CC-Di-B_RRS were produced in the same manner. Annotated amino acid sequences for all fusion proteins are provided in the Supplementary Information and a list of primers used is provided in Supplementary Table 1.

### Expression and purification of CC-Di-A_S-mCitrine-H10

pT7-CC-Di-A_S-mCitrine-H10 was transformed into chemically competent BL21(DE3)pLys cells (Novagen/Merck Millipore), which were subsequently plated onto LB-agar under carbenicillin and chloramphenicol antibiotic selection and incubated overnight at 37 °C. A single colony was selected to inoculate 20 mL of LB containing 50 µg mL^–1^ carbenicillin and 34 µg mL^–1^ chloramphenicol. This starter culture was grown overnight (18 h) at 30 °C with shaking. The following morning, 1 L of Terrific Broth (TB) containing 50 µg mL^–1^ carbenicillin and 34 µg mL^–1^ chloramphenicol was inoculated with 20 mL of starter culture and grown at 37 °C with shaking until it reached OD_600_ = 0.6. The culture was then transferred to another shaker-incubator at 18 °C and grown for 20 min before induction with 1 mM isopropyl-β-d-1-thiogalactoside (IPTG). The induced culture was then grown overnight (18 h) at 18 °C with shaking. Cells were harvested by centrifugation at 5000 × *g* and 4 °C for 30 min in a TLA8.1000 rotor (Beckman Coulter). The supernatant was discarded and the resulting cell pellet recovered and frozen at −20 °C until required.

To extract and purify the protein, the frozen cell pellet was thawed on ice, then suspended in 20 mL lysis buffer (50 mM Tris.HCl, pH 7.5, 500 mM NaCl, 1 mM dithiothreitol (DTT), 10 mM imidazole, and 0.1% (v/v) Triton X-100). In total, 7500 U of *Serratia marcescens* DNAse (SmDNAse, Biochemistry core facility of the Max Planck Institute of Biochemistry) and 1 mg mL^–1^ (final concentration) lysozyme from chicken egg white were added to the cell suspension, which was then incubated on ice for 30 min. The lysed cells were sonicated in a water-ice bath using a Branson Sonifier 250D, with 30 s pulses of 40% amplitude, followed by 30 s periods of 0% amplitude (eight cycles were performed). The lysed cells were then clarified by centrifugation at 40,000 × *g* and 4 °C for 45 min in a JA25.50 rotor (Beckman Coulter). Following centrifugation, the supernatant was retained as the cleared lysate.

To purify CC-Di-A_S-mCitrine-H10, 1 mL Ni-NTA Superflow resin (Qiagen) was equilibrated in binding buffer (50 mM Tris.HCl, pH 7.5, 500 mM NaCl, 1 mM DTT, 10 mM imidazole, and 0.1% (v/v) Triton X-100). The equilibrated resin was then added to the cleared lysate and the mixture was incubated at 4 °C for 1 h. The mixture was then placed into a disposable glass column (Bio-Rad) and the flow-through collected. The resin was then washed with 3 × 10 mL of wash buffer (50 mM Tris.HCl, pH 7.5, 500 mM NaCl, 1 mM DTT, 20 mM imidazole). After washing, protein was eluted using 10 × 0.5 mL elution buffer (35 mM Tris.HCl, pH 7.5, 350 mM NaCl, 0.7 mM DTT, 300 mM imidazole). Fractions were analyzed by SDS-PAGE and UV-Vis spectroscopy.

Peak fractions were pooled and further purified by size exclusion chromatography using a HiLoad 16/600 Superdex 75 pg column (GE Healthcare) equilibrated in storage buffer (50 mM Tris.HCl, pH 7.5, 100 mM NaCl, 1 mM DTT, 10% glycerol). The column was run isocratically with a flow rate of 0.5 mL min^–1^ using an Äkta Pure chromatography system (GE Healthcare). Peak fractions were analyzed by SDS-PAGE and UV-Vis spectroscopy, pooled, aliquoted, and flash frozen in liquid nitrogen before being stored at −80 °C. The purified protein was further analyzed by LC-MS to confirm correct mass and purity (expected mass: 34,690.5586; observed mass: 34669.3394). Protein concentration was determined by fluorescence absorbance of the mCitrine label (*ɛ*_515_(mCitrine) = 69,000 M^–1^ cm^–1^).

### Expression of hybSnf7(1–13)-mVenus-H6-CC-Di-A_S and hybSnf7(1–13)-mApple-H6-CC-Di-B_RRS

pT7-hybSnf7(1–13)-mVenus-H6-CC-Di-A_S and pT7-hybSnf7(1–13)-mApple-H6-CC-Di-B_RRS were each transformed into chemically competent BL21(DE3)pLysS cells and expression cultures were grown to OD_600_ = 1.0 and then induced under the same conditions as above. The induced cultures were grown overnight (18 h) at 18 °C with shaking. Cells were harvested and stored as above.

### Purification of hybSnf7(1–13)-mVenus-H6-CC-Di-A_S

The frozen cell pellet as described above was thawed on ice then suspended in 25 mL lysis buffer (as above). In total, 7500 U of SmDNAse, 1 mg mL^–1^ (final concentration), and a protease inhibitor cocktail tablet (Roche cOmplete EDTA-free) were added to the cell suspension, which was then incubated on ice for 30 min. The lysed cells were sonicated and clarified as above.

hybSnf7(1–13)-mVenus-H6-CC-Di-A_S was purified from the cleared lysate using Ni-NTA affinity chromatography as described above. Peak fractions were pooled and concentrated (Amicon Ultra-15 with 10 kDa NMWL), before being further purified by size exclusion chromatography using a Superdex 75 10/300 GL column (GE Healthcare) equilibrated in storage buffer (50 mM Tris.HCl, pH 7.5, 100 mM NaCl, 1 mM DTT, and 10% glycerol). The column was run isocratically with a flow rate of 0.4 mL min^–1^ using an Äkta Pure chromatography system (GE Healthcare). The protein eluted as two peaks, with the first at 10.8 mL corresponding to the full-length protein and the second at 11.5 mL corresponding to the truncated protein, as confirmed by SDS-PAGE and LC-MS. The 10.8-mL peak was collected, aliquoted, and flash frozen in liquid nitrogen before being stored at −80 °C. The observed mass obtained by LC-MS was 34,792.8122 (expected mass: 34,793.8477). Protein concentration was determined by fluorescence absorbance of the mVenus label (*ε*_515_(mVenus) = 104,000 M^–1^ cm^–1^).

### Purification of hybSnf7(1–13)-mApple-H6-CC-Di-B_RRS

The cleared lysate was prepared in the same manner as for hybSnf7(1–13)-mVenus-H6-CC-Di-A_S. To purify hybSnf7(1–13)-mApple-H6-CC-Di-B_RRS from the cleared lysate, 1 mL Ni-NTA Superflow resin (Qiagen) was equilibrated in binding buffer (50 mM Tris.HCl, pH 7.5, 500 mM NaCl, 1 mM DTT, 10 mM imidazole, 0.1% (v/v) Triton X-100). The equilibrated resin was then added to the cleared lysate and the mixture was incubated at 4 °C for 1 h. The mixture was then placed into a disposable glass column (Bio-Rad) and the flow-through collected. The resin was then washed with 3 × 10 mL of high salt wash buffer (50 mM Tris.HCl, pH 7.5, 1 M NaCl, 1 mM DTT, 20 mM imidazole), followed by 2 × 10 mL of wash buffer (50 mM Tris.HCl, pH 7.5, 500 mM NaCl, 1 mM DTT, 20 mM imidazole). After washing, protein was eluted using 8 × 0.5 mL elution buffer (35 mM Tris.HCl, pH 7.5, 350 mM NaCl, 0.7 mM DTT, 300 mM imidazole). Fractions were analyzed by SDS-PAGE and UV-Vis spectroscopy.

Peak fractions were pooled before being further purified by ion exchange chromatography. The pooled fractions were diluted 10-fold into Buffer A (50 mM Tris.HCl, pH 7.5, 50 mM NaCl, 1 mM DTT). The diluted protein was then loaded onto a 1-mL HiTrap SP HP column (GE Healthcare) that had been equilibrated in Buffer A. The small fraction of truncated protein present did not bind the column and was discarded in the flow-through. The column was washed with 10 mL Buffer A and then protein was eluted by applying a gradient of 0–50% Buffer B (50 mM Tris.HCl, pH 7.5, 1 M NaCl, 1 mM DTT) over 20 column volumes. The full-length protein eluted at ~12% Buffer B. Peak fractions were collected and pooled and analyzed by SDS-PAGE and LC-MS. The observed mass was 34,884.0274 (expected: 34,885.5898). Sterile 50% glycerol was added to the pooled purified protein to a final concentration of 10% glycerol. The protein was then aliquoted and flash frozen in liquid nitrogen before being stored at –80 °C. Protein concentration was determined by fluorescence absorbance of the mApple label (*ε*_568_(mApple) =  75,000 M^–1^ cm^–1^).

### Circular dichroism spectroscopy

Circular dichroism spectroscopy was performed on JASCO J-715 and J-810 spectropolarimeters (JASCO) fitted with Peltier temperature controllers. Peptides were dissolved in PBS buffer and measured in quartz cuvettes with 1 mm pathlength.

Thermal denaturation experiments were performed with a 5–90 °C temperature ramp at a rate of 40 °C h^–1^. Full spectra were determined at 5 °C intervals and CD at 222 nm was measured at 1 °C intervals (1 nm interval, 1 nm bandwidth, 16 s response). *T*_m_ values were determined from the second derivative of the thermal denaturation curve.

Phosphorylation reactions were performed in PBS with the addition of 10 mM MgCl_2_ and 1 mM ATP. PKA (2500 U) was added to a reaction volume of 300 µL to initiate the reaction. CD at 222 nm was measured over time for the duration of the reaction (10 s intervals). Dephosphorylation reactions were performed in 50 mM Tris.HCl, pH 7.5, 150 mM NaCl, with peptide concentrations of 10 µM each. To begin dephosphorylation, 1 mM MnCl_2_ and 4000 U lambda protein phosphatase were added to the reaction volume of 300 µL.

### Analytical HPLC

HPLC analysis of peptides was performed on an Agilent 1260 Infinity II HPLC System (Agilent Technologies) using a Kinetex C18 column (150 × 3 mm, 2.6 µm beads, Phenomenex Inc.). A gradient of water/0.1% TFA to acetonitrile/0.08% TFA (15% acetonitrile/0.08% TFA from 0 to 2 min, 15–60% from 2 to 45 min, and 60–90% from 45 to 60 min) was used at 420 µL/min flowrate, with UV detection at 214 nm. Injection volume was 20 µL. Agilent Open LAB CDS Chemstation Edition (software revision C.01.08) was used for data integration and analysis. Chromatograms were processed (baseline subtraction and normalization) and plotted in a Jupyter notebook.

Phosphorylation reactions were performed in PBS with the addition of 10 mM MgCl_2_ and 1 mM ATP. PKA (5000 U) was added to a reaction volume of 600 µL to initiate the reaction. The reaction temperature was maintained at 25 °C throughout. In all, 60 µL aliquots were taken at specified time intervals, to which 20 mM EDTA was added (final concentration) to stop the reaction. Aliquots were stored at −20 °C until required.

### Supported lipid bilayer experiments

Supported lipid bilayers (SLBs) were formed by the fusion of small unilamellar vesicles (SUVs) on glass coverslips (Menzel #1.5, 24 × 24 mm). The coverslips were cleaned by washing in ultrapure water, followed by absolute ethanol before drying under a stream of nitrogen. Once dry, the coverslips were cleaned in an oxygen plasma cleaner (0.3 mbar O_2_, 50% power for 30 s, Diener Zepto, and Diener electronic GmbH). A buffer reservoir was formed above the coverslips by attaching the tubular part of 0.5 mL microcentrifuge tubes to the glass surface with optical adhesive (Norland Optical Adhesive 68, Norland Products). To prepare SUVs, lipids (DOPC with 2 mol% DGS-NTA(Ni) (Avanti Polar Lipids, Inc.) and 0.1 mol% ATTO655-DOPE (ATTO-TEC GmbH)) were dissolved and mixed in chloroform and then dried under a gentle stream of nitrogen before drying under vacuum for 1 h. The dried lipid film was rehydrated in SLB buffer (50 mM Tris.HCl pH 7.5, 300 mM KCl, and 5 mM MgCl_2_) at a concentration of 1 mg mL^–1^, before being vortexed and sonicated (bath sonicator, Branson) until clear. To form SLBs, the prepared SUVs were diluted to 0.1 mg mL^–1^ in 200 µL SLB buffer and placed atop the prepared glass coverslip surface in the buffer reservoir. CaCl_2_ was added to a final concentration of 3 mM to promote SUV fusion with the glass surface. After 30 min incubation at room temperature, the glass surface was washed with 10 × 200 µL SLB buffer to remove unfused SUVs. SLBs were passivated by incubation with 1 mg mL^–1^ bovine serum albumin (BSA) for 1 h at room temperature to reduce non-specific binding. This concentration was then reduced to 0.4 mg/mL for subsequent experimental steps.

To couple anchor protein (DiA_S-mCitrine-H10) to SLBs, SLBs were incubated with 1 µM DiA_S-mCitrine-H10 for 30 min. Cargo peptide (Alexa 594-Di-B_RRS) was then added to a final concentration of 1 µM.

To detach cargo peptide, MgATP (10 mM MgCl_2_, 1 mM ATP, final concentrations) was added to the SLBs followed by 500 U protein kinase A (PKA/cAMP-dependent protein kinase, New England Biolabs). To re-attach cargo peptide, 1 mM MnCl_2_ (final concentration) and 400 U lambda protein phosphatase (λPP) were added to SLBs.

For experiments with hybSnf7(1–13)-mVenus-H6-DiA_S and hybSnf7(1–13)-mApple-H6-Di-B_RRS, SLBs were prepared as above, but with the SLBs comprising DOPC (Avanti Polar Lipids, Inc.) with 0.1 mol% ATTO655-DOPE (ATTO-TEC GmbH). For FRET experiments, the ATTO655-DOPE was omitted. Proteins were then added individually or together at 1 µM final concentration. To detach proteins, MgATP (10 mM MgCl_2_, 1 mM ATP, final concentrations) was added to the SLBs followed by 500 U protein kinase A (PKA/cAMP-dependent protein kinase, New England Biolabs). To re-attach proteins, 1 mM MnCl_2_ (final concentration) and 400 U lambda protein phosphatase (λPP) were added to SLBs.

### Microscopy and image analysis

Imaging was performed on a Zeiss LSM780 confocal microscope using a Zeiss C-Apochromat 40X/1.20 water-immersion objective (Carl Zeiss), and a Zeiss LSM800 confocal microscope also using a Zeiss C-Apochromat 40X/1.20 water-immersion objective. mCitrine and mVenus were excited at 488 nm, mApple at 561 nm, and ATTO 655 at 633 nm (or 640 nm for the LSM800). In the case of multi-color imaging, each color was excited using separate imaging tracks to avoid cross-talk. For the measurement of stimulated emission by FRET, excitation was performed at 488 nm and emission was measured with a 600-nm high-pass filter. Images were obtained using a pinhole diameter of 1 Airy unit, a resolution of 512 × 512 pixels, and a scan rate of 1.58 µs (LSM780) or 1.03 µs (LSM800) per pixel. The temperature at the microscope stage was 21 °C.

To determine the degree of membrane localization, z-stacks of images (where z is the axis perpendicular to the plane of the SLB) were obtained by confocal microscopy. Image analysis was performed using Fiji^63^ Where orthogonal xz plane images are presented, the brightness and contrast of these images has been adjusted uniformly to aid visibility.

For the analysis of SLB membrane-localized fluorescence intensity over time, time series of z-stacked images were analyzed using a custom ImageJ macro (available at https://github.com/leonharrington/zanalyzer). At each time point, the macro generates a z-profile from the mean fluorescence intensity from each constituent image of the z-stack. The peak intensity value from each profile is then plotted against time to give the resulting fluorescence intensity time course for the membrane-localized fraction.

To quantitate membrane localization, peak areas were calculated by integrating the peaks of z-profiles generated from confocal microscopy images in OriginPro 2018. The Peak Analyzer function was used with manual baseline subtraction to correct for the fluorescence from the bulk solution of the SLB.

### Kinetic analysis

Data plotting and fitting was performed with Graphpad Prism 8. For the HPLC experiments, reaction curves were generated by plotting integrated peak area versus reaction time. The curves were then fitted by non-linear regression to the equations:

2 $$y = y_0 + \left( {y_\infty - y_0} \right)\left( {1 - e^{ - kx}} \right)$$

and

3 $$y = y_0 \cdot e^{ - kx}$$

for product formation and substrate consumption, respectively. Fitted values are reported for three independent experiments.

Reaction curves obtained in CD and SLB experiments were fitted by non-linear regression to Eq. (2). Fitted values are reported as mean  ± standard deviation from the indicated number of experimental replicates.

### Reporting summary

Further information on research design is available in the Nature Research Reporting Summary linked to this article.

## Supplementary information

Supplementary Information

Reporting Summary

## Data Availability

The datasets generated during and/or analyzed during the current study are available from the corresponding author on reasonable request.

## Code availability

The custom ImageJ macro used to analyze SLB membrane-localized fluorescence intensity over time from time series of z-stacked images is available at https://github.com/leonharrington/zanalyzer (DOI: 10.5281/zenodo.4495771) or from the corresponding author on request.
