# Supplementary material for: De novo design of a reversible phosphorylation-dependent switch for membrane targeting
Source: Nat Commun. 2021 Mar 5;12:1472. doi: 10.1038/s41467-021-21622-5 (PMC7935970; doi:10.1038/s41467-021-21622-5)
Supplement: Supplementary file 1 — Supplementary Information [file 41467_2021_21622_MOESM1_ESM.pdf]

De novo design of a reversible phosphorylation-dependent switch for membrane targeting

Supplementary Information

Leon Harrington[1], Jordan M. Fletcher[2], Tamara Heermann[1], Derek N. Woolfson[2,3,4*], Petra Schwille[1,*]

[1]Department of Molecular and Cellular Biophysics, Max Planck Institute of Biochemistry, Martinsried, 92152, Germany

[2]School of Chemistry, University of Bristol, Cantock's Close, Bristol, BS8 1TS, United Kingdom

[3]School of Biochemistry, University of Bristol, Medical Sciences Building, University Walk, Bristol, BS8 1TD, United Kingdom

[4]Max Planck-Bristol Centre for Minimal Biology, University of Bristol, Cantock's Close, Bristol, BS8 1TS, United Kingdom

*Correspondence should be addressed to Petra Schwille (schwille@biochem.mpg.de) or Derek N. Woolfson (d.n.woolfson@bristol.ac.uk)

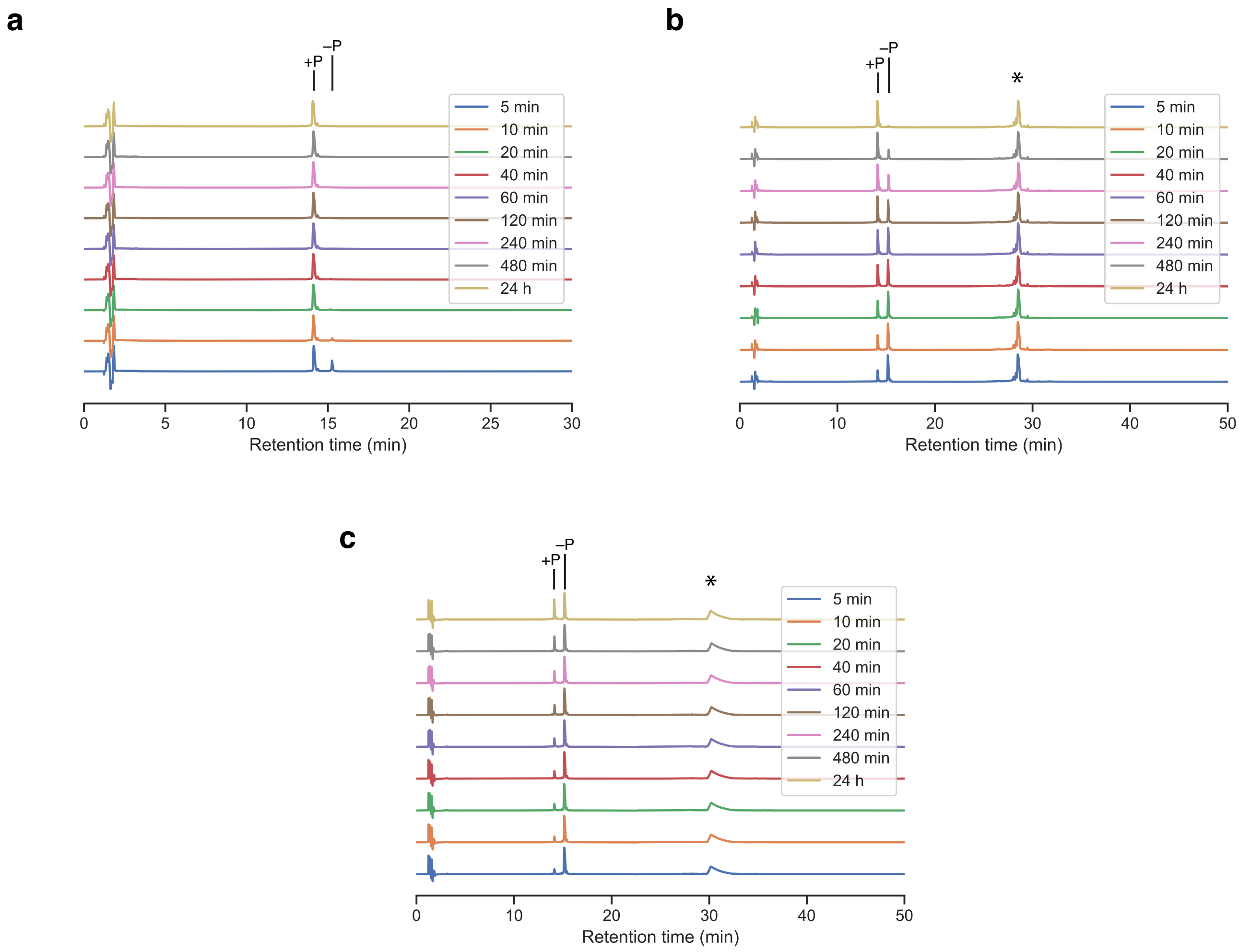

Supplementary Figure 1. Kinetics of CC-Di-B_RRS phosphorylation. Representative HPLC chromatograms are shown for samples taken from phosphorylation reactions at the indicated time intervals with (**a**) CC-Di-B_RRS only, (**b**) CC-Di-B_RRS:CC-Di-A_S, and (**c**) CC-Di-B_RRS:CC-Di-A_N^4. The peak corresponding to CC-Di-A_S/CC-Di-A_N^4 is indicated by an asterisk. Each peptide was present at a concentration of 50 μM. The reaction buffer was PBS with the addition of 10 mM $MgCl_2$ and 1 mM ATP. PKA (5,000 U) was added to a reaction volume of 600 μL to initiate the reaction. The reaction temperature was maintained at 25 °C throughout. Experiments were performed in triplicate.

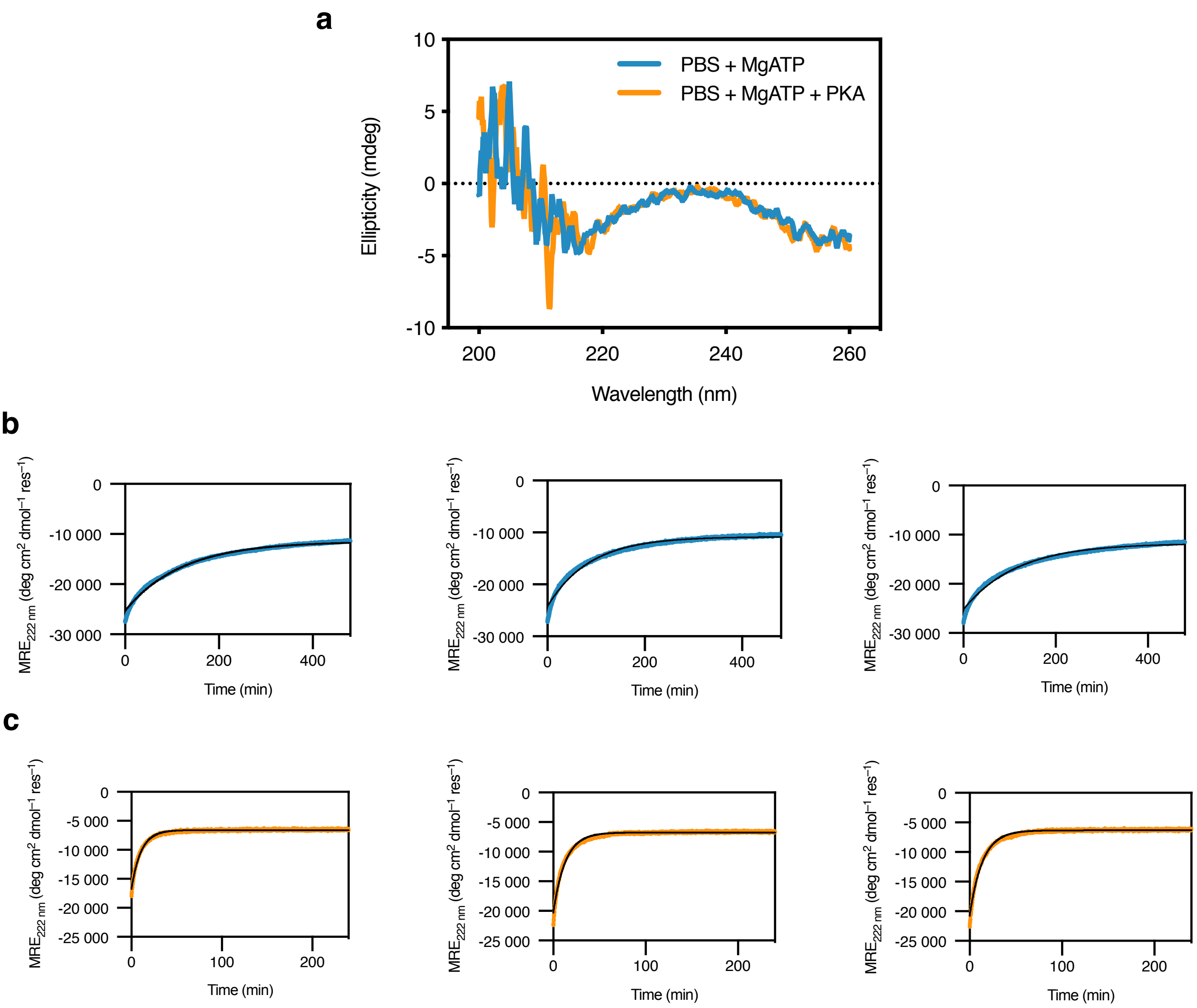

Supplementary Figure 2. (**a**) **Quantity of PKA used does not contribute background CD signal.** CD spectra are shown for the kinase reaction buffer (PBS + 10 mM $MgCl_2$ and 1 mM ATP) with and without the addition of PKA (2,500 U). **Kinetic plots of phosphorylation-induced dissociation of CC-Di-A_S:CC-Di-B_RRS at** (**b**) **25 °C and** (**c**) **35 °C.** Data were fitted to the integrated form of a first-order rate equation by non-linear regression.

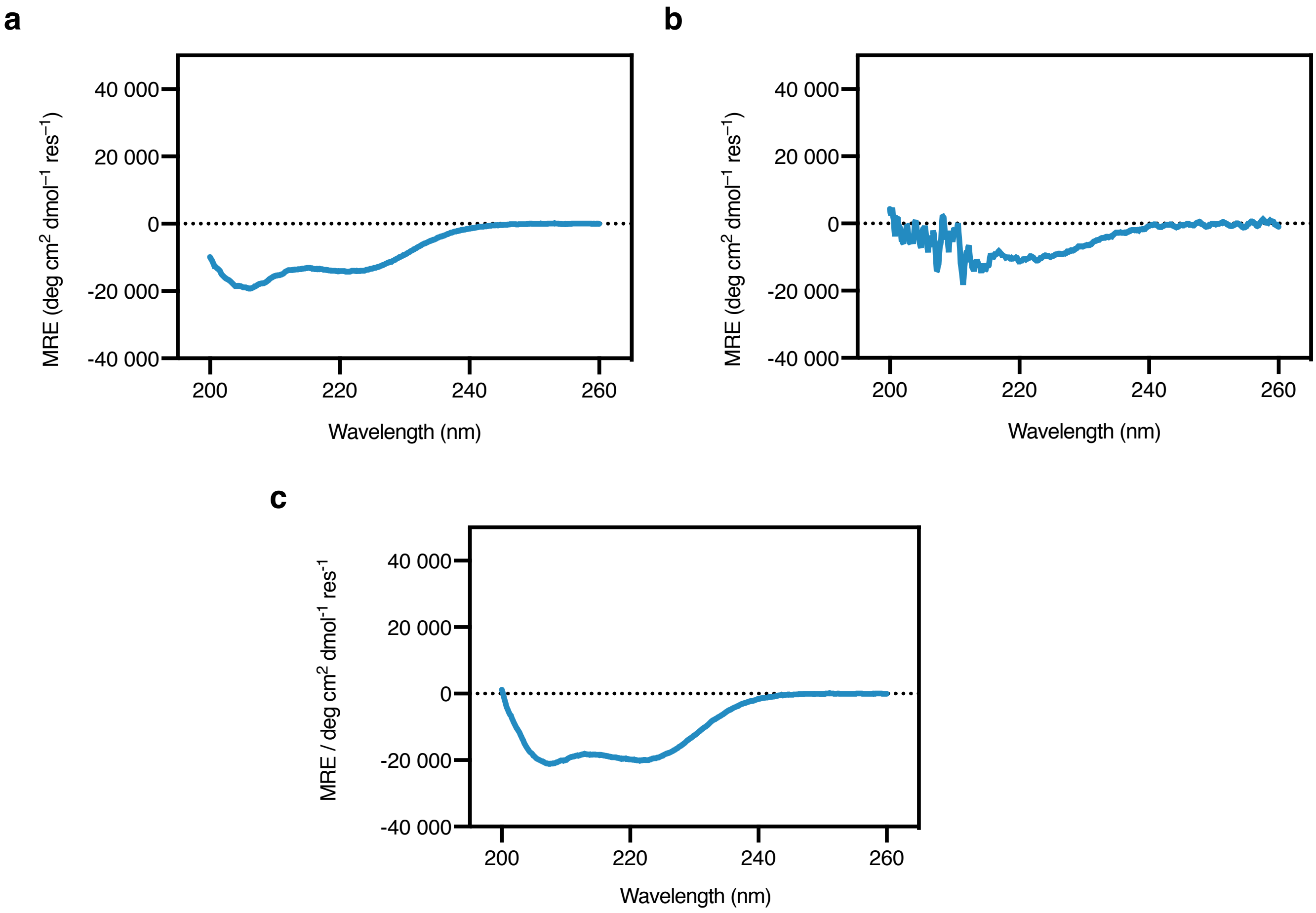

Supplementary Figure 3. CD spectra of heterodimers with CC-Di-B_RRpS as the partner peptide. (**a**) CD spectrum of CC-Di-A_S with synthetically prepared CC-Di-B_RRpS. (**b**) CD spectrum of CC-Di-A_S and CC-Di-B_RRS, where enzymatic phosphorylation had been allowed to reach completion. (**c**) CD spectrum of CC-Di-A$_N$[4] and synthetically prepared CC-Di-B_RRpS. Individual peptide concentrations were 50 μM. The buffer for (**a**) and (**c**) was PBS; for (**b**) it was PBS with 10 mM $MgCl_2$ and 1 mM ATP.

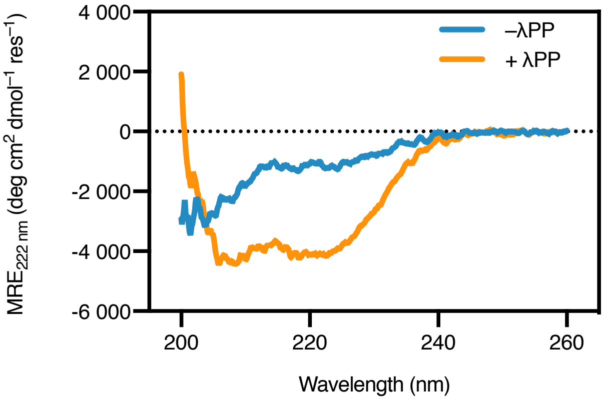

Supplementary Figure 4. CD spectra of CC-Di-A_S with CC-Di-B_RRpS before and after dephosphorylation. CD spectra are shown for CC-Di-A_S and CC-Di-B_RRpS before and after the addition of 1 mM $MnCl_2$ and 4000 U lambda protein phosphatase. Individual peptide concentrations were 10 µM. A reduced concentration was used as we found that at 50 µM the addition of $MnCl_2$ caused precipitation of peptide, possibly due to Mn^{2+}-mediated cross-linking of CC-Di-B_RRpS. The reaction buffer was 50 mM Tris.HCl, pH 7.5, 150 mM NaCl.

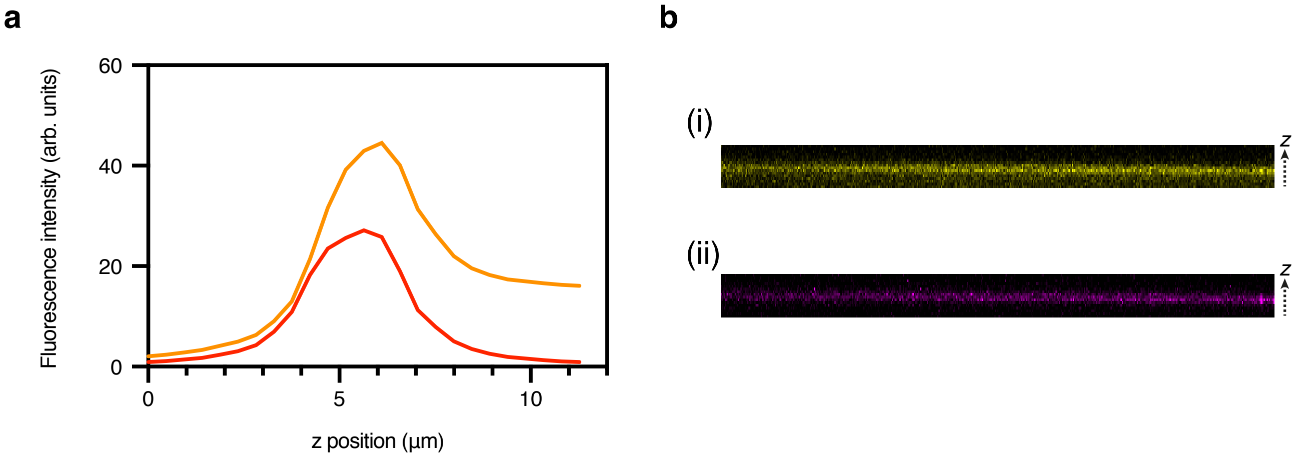

Supplementary Figure 5. Attachment of anchor molecule to SLB. (**a**) z-profile fluorescence intensity plots are shown for the channels corresponding to the lipid bilayer (via ATTO655-DOPE, red) and the anchor molecule (CC-Di-A_S-mCitrine-H10, orange). Profiles were generated from z-stacks of confocal microscopy images. (**b**) Orthogonal views of the xz plane for the z-stack of images used in **a** for (**i**) CC-Di-A_S-mCitrine-H10, and (**ii**) ATTO655-DOPE. The brightness and contrast were adjusted uniformly to aid visibility. The SLB was prepared as described in Materials and Methods (composition: DOPC with 2 mol% DGS-NTA(Ni^{2+}) and 0.1 mol% ATTO655-DOPE) and incubated with 1 µM CC-Di-A_S-mCitrine-H10. The buffer was 50 mM Tris.HCl, pH 7.5, 300 mM KCl, and 5 mM $MgCl_2$.

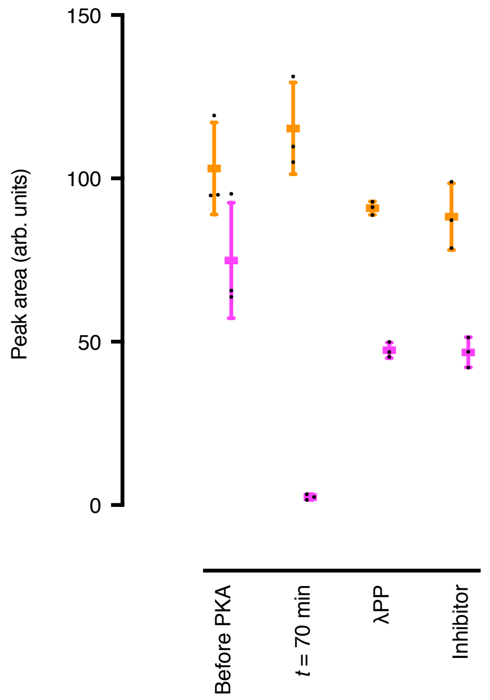

Supplementary Figure 6. Presence of kinase after dephosphorylation does not appreciably alter membrane binding. The experiments of Fig. 3 were repeated with an additional step, wherein 1 µM of the PKA inhibitor H89 dihydrochloride hydrate (N-[2-(p-bromocinnamylamino)ethyl]-5-isoquinolinesulfonamide dihydrochloride; K_i = 48 nM) was added after dephosphorylation by lambda protein phosphatase (λPP) and $MnCl_2$. The membrane binding of CC-Di-A_S-Citrine-H10 (orange) and AF594-CC-Di-B_RRS (pink) was quantitated from fluorescence z-profiles in the same manner as Fig. 3 (*n* = 3; error bars represent mean ± S.D.) SLBs were DOPC with 2 mol% DGS-NTA(Ni^{2+}) and 0.1 mol% ATTO655-DOPE. AF594-CC-Di-B_RRS and CC-Di-A_S-mCitrine-H10 were both at 1 µM. The SLB buffer was 50 mM Tris.HCl, pH 7.5, 300 mM KCl, and 5 mM $MgCl_2$. Experiments were performed on an LSM800 confocal microscope as detailed in Materials and Methods. The Alexa 594 fluorophore (AF594) was excited with a 561 nm diode laser. Experiments were performed in triplicate. No significant change in membrane binding was observed after the addition of H89 ($p > 0.05$ for both SwitchA and SwitchB; t = 0.4401 and 0.1973 respectively; unpaired two-tailed *t* test).

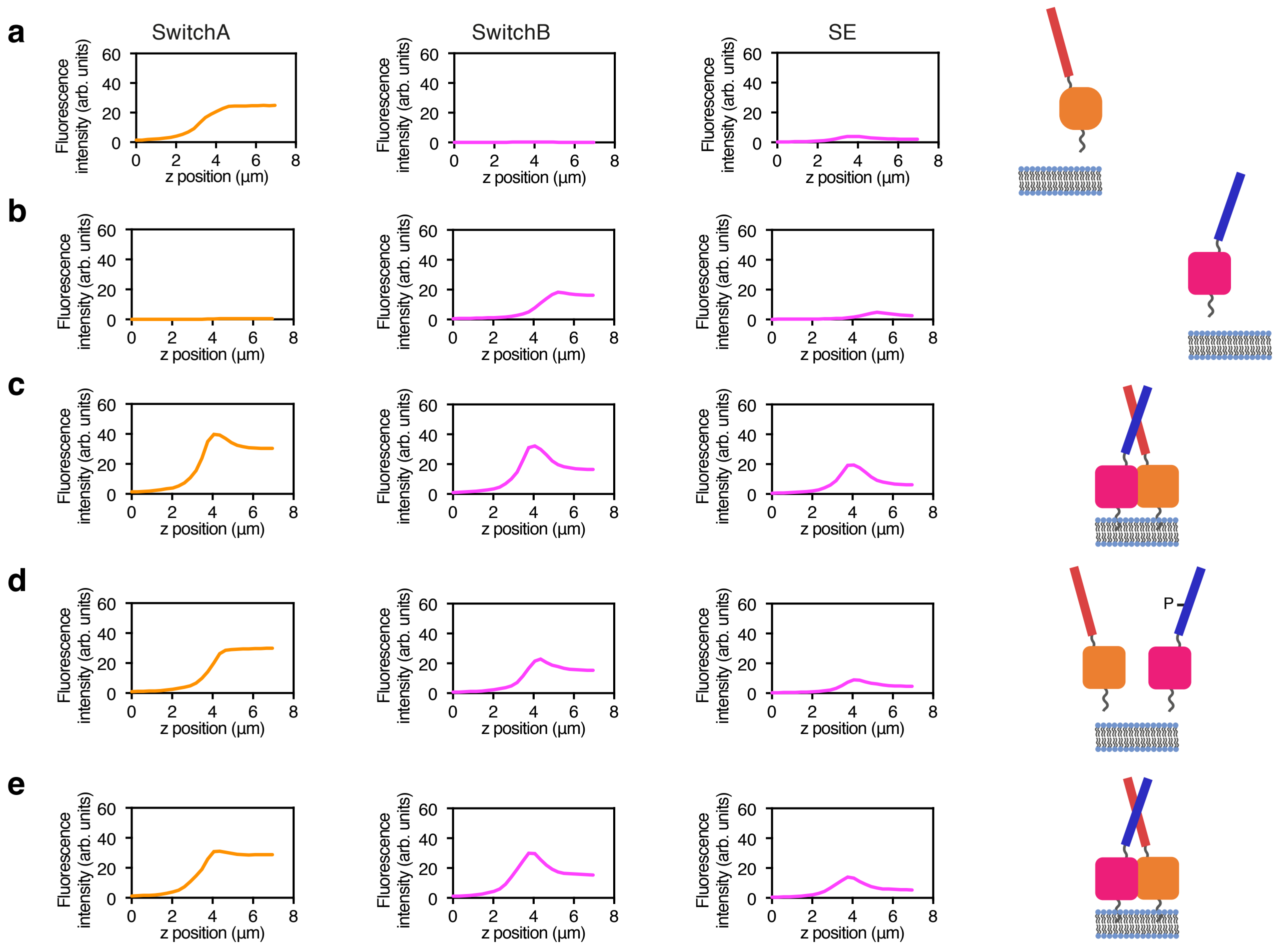

Supplementary Figure 7. Switching of membrane binding state followed by FRET. Representative fluorescence intensity z-profiles obtained from confocal microscopy of SLBs and illustrative schematics are shown for: (**a**) 1 µM SwitchA only, (**b**) 1 µM SwitchB only, (**c**) 1 µM SwitchA and 1 µM SwitchB, (**d**) 1 µM SwitchA and 1 µM SwitchB after phosphorylation for 70 min, (**e**) the reaction of **d** after dephosphorylation. Z-profiles are shown for the SwitchA (mVenus), SwitchB (mApple) and SE (stimulated emission) imaging channels. SLBs were composed of DOPC. The starting SLB buffer was 50 mM Tris.HCl, pH 7.5, 300 mM KCl and 5 mM $MgCl_2$.

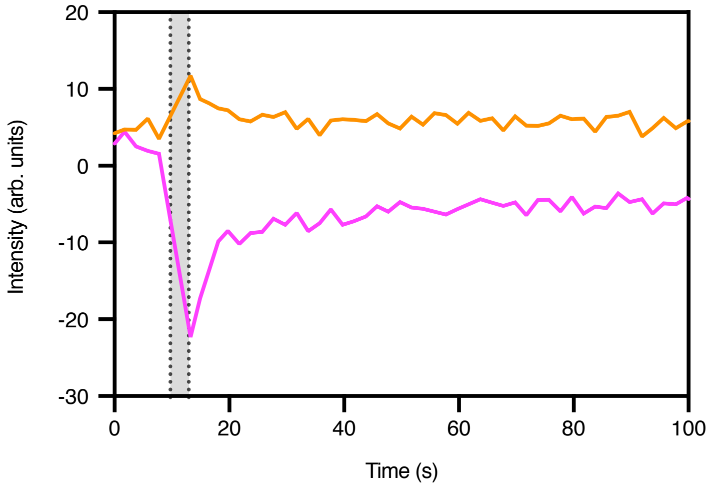

Supplementary Figure 8. Acceptor photobleaching of membrane-bound SwitchA:SwitchB. Donor (SwitchA/mVenus, orange line) and acceptor (SwitchB/mApple, pink line) fluorescence was monitored before bleaching the acceptor with the 561 nm laser during the period indicated by the grey-shaded area. Fluorescence intensity was then monitored to observe recovery. Enhancement of donor fluorescence was observed upon acceptor photobleaching, indicative of FRET. SLBs were composed of DOPC. The SLB buffer was 50 mM Tris.HCl, pH 7.5, 300 mM KCl and 5 mM $MgCl_2$. SwitchA and SwitchB were both present at 1 µM each. The experiment was performed in triplicate; one representative fluorescence time course is shown.

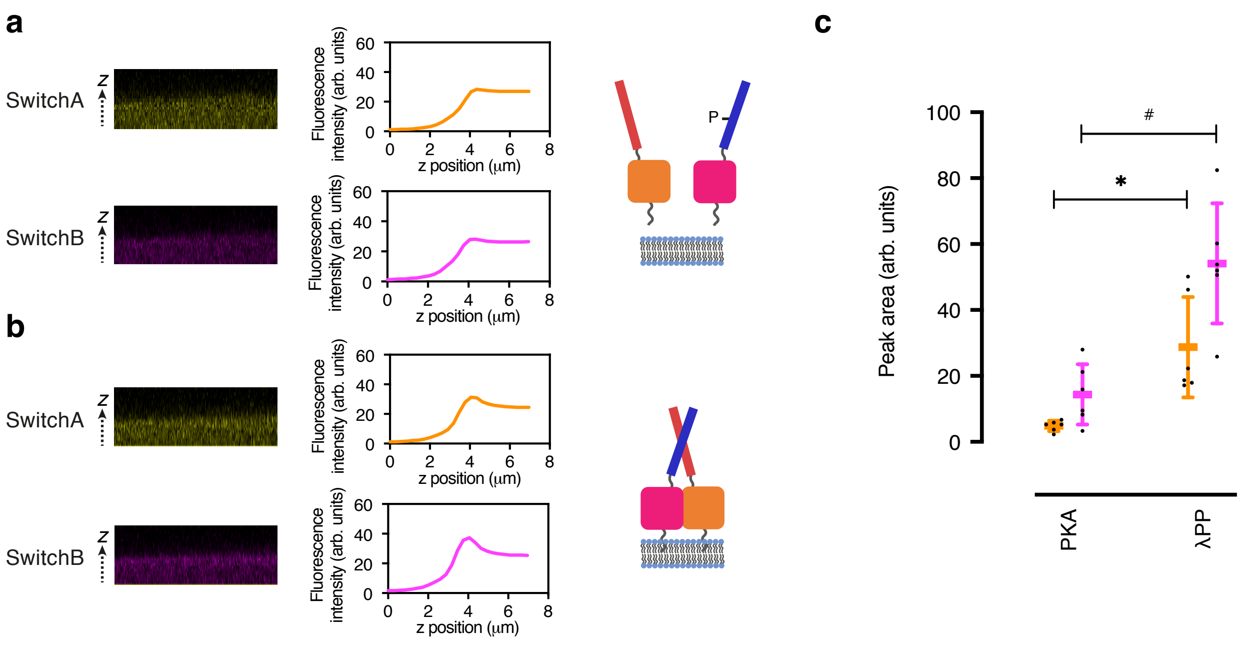

Supplementary Figure 9. Recovery of membrane binding after dephosphorylation. Representative orthogonal views of the xz plane of confocal microscopy image z-stacks are shown together with representative fluorescence intensity z-profiles and illustrative schematics for: (**a**) 1 µM each of SwitchA and SwitchB together on an SLB to which MgATP and PKA has been added and phosphorylation allowed to occur for 1h (as also shown in Fig. 4e), (**b**) the reaction of **a**, to which λPP and $MnCl_2$ were added. (**c**) Quantitation of membrane binding of SwitchA (orange) and phosphorylated/dephosphorylated SwitchB (pink) as described in **a** and **b**. Peak areas were calculated from z-profiles, as shown in **a** and **b** (n = 3 independent experiments; error bars represent mean ± S.D; ✱ p = 0.0018, t = 4.2155; # p = 0.0012, t = 4.4782; unpaired two-tailed t test). SLBs were composed of DOPC with 0.1 mol% ATTO655-DOPE. The reaction buffer was 50 mM Tris.HCl, pH 7.5, 300 mM KCl, 5 mM $MgCl_2$, and 1 mM ATP.

Fusion protein sequences

CC-Di-A_S-mCitrine-H10

MGSEIAALEQESAALEKENAALEWEIAALEQGSGSGSTMGSSGSGSSGSSGLVPRGSHMVSKGEELFTGVVP
ILVELDGDVNGHKFSVSGEGEGDATYGKLTLKFICTTGKLPVPWPTLVTTFGYGLMCFARYPDHMKQHDFFK
SAMPEGYVQERTIFFKDDGNYKTRAEVKFEGDTLVNRIELKGIDFKEDGNILGHKLEYNYNSHNVYIMADKQ
KNGIKVNFKIRHNIEDGSVQLADHYQQNTPIGDGPVLLPDNHYLSYQSKLSKDPNEKRDHMVLLEFVTAAGI
TLGMDELYKTSGSGSSGSGHHHHHHHHHH

CC-Di-A_S: blue, mCitrine: orange, H10: green.

hybSnf7(1–13)-mVenus-H6-CC-Di-A_S

MSVWGKLFGWGGGTSGSGSSRGRSMVSKGEELFTGVVPILVELDGDVNGHKFSVSGEGEGDATYGKLTLKLI
CTTGKLPVPWPTLVTTLGYGLQCFARYPDHMKQHDFFKSAMPEGYVQERTIFFKDDGNYKTRAEVKFEGDTL
VNRIELKGIDFKEDGNILGHKLEYNYNSHNVYITADKQKNGIKANFKIRHNIEDGGVQLADHYQQNTPIGDG
PVLLPDNHYLSYQSKLSKDPNEKRDHMVLLEFVTAAGITLGMDELYKGSGSGSTMGSSHHHHHHSSGLVPRG
SEIAALEQESAALEKENAALEWEIAALEQG

hybSnf7(1–13): bold, mVenus: orange, H6: green, CC-Di-A_S: blue.

hybSnf7(1–13)-mApple-H6-CC-Di-B_RRS

MSVWGKLFGWGGGTSGSGSSRGRSMVSKGEENNMAIIKEFMRFKVHMEGSVNGHEFEIEGEGEGRPYEAFQT
AKLKVTKGGPLPFAWDILSPQFMYGSKVYIKHPADIPDYFKLSFPEGFRWERVMNFEDGGIIHVNQDSSLQD
GVFIYKVKLRGTNFPSDGPVMQKKTMGWEASEERMYPEDGALKSEIKKRLKLKDGGHYAAEVKTTYKAKKPV
QLPGAYIVDIKLDIVSHNEDYTIVEQYERAEGRHSTGGMDELYKGSGSGSTMGSSHHHHHHSSGLVPRGSKI
AALRRKSAALKYKNAALKKKIAALKQG

hybSnf7(1–13): bold, mApple: pink, H6: green, CC-Di-B_RRS: red.

Primer List

Name	Sequence
LH77	**TTCGGTGTACGTTCCTAA**CCTAGGTTTGGATCCGGCTGC
LH76	**TTCCAGGGCTGCGATCTC**GCTACCCATATATCTCCTTCTTAAAG
LH78	AGAAGGAGATATACCATGGGTAGCGAGATCGCAGCCC
LH70	AGCCGGATCCAAGCTTCATTAATGGTGATGGTGATGGTGGTG
LH38	AAGCTTGGATCCGGCTGCTAACAAAGCCCG
LH40	CGGATCCAAGCTTCATTATTTGTACAGTTCGTCCATGCCCAG
SC47	CAGAAGTGGTCCTGCAACTTTAT
SC46	ATAAAGTTGCAGGACCACTTCTG
LH120	**CTCTCTTCGGTTGGACCT**CTTCTAACGCGtaatgaagcttggatccggc
LH121	**AGGTCCAACCGAAGAGAG**AAGACCACATcgaacgtccgcgactcgaac
LH137	TGGGGTAAACTGTTCGGTTGGGGTGGTGGTACCTCTGGCTCTG
LH138	TGGGGTAAACTGTTCGGTTGGGGTGGTGGTACCAGCGGTTCCG
LH139	CCAACCGAACAGTTTACCCCAAACAGACATGGTATATCTCCTTCTTAAAG

Supplementary Table 1: List of primers used in this work
